# Quick screening for *Brucella* virulence genes with the Bat-VFDB: *Brucella* addition to the Virulence Factor DataBase

**DOI:** 10.1128/mra.00730-25

**Published:** 2025-10-24

**Authors:** Andries A. Kampfraath, Jeroen Koomen, Timo Dijkstra, Ad Koets, Marielle H. van den Esker

**Affiliations:** 1 Wageningen Bioveterinary Research (WBVR), Wageningen University & Research 4508, Lelystad, the Netherlands; Indiana University, Bloomington, Bloomington, Indiana, USA

**Keywords:** virulence genes, database, brucellosis, genome screening

## Abstract

The Bat-VFDB database was developed to enable quick screening for, and identification of, virulence genes in genomes of *Brucella* species. This database comprises a complete set of known *Brucella* spp. virulence genes and can be used by the ABRicate tool for screening large sets of genomes at once.

## ANNOUNCEMENT

Brucellosis is an infectious zoonosis with millions of people affected annually. The disease is caused by facultative intracellular bacteria of the *Brucella* genus. The core of this genus consists of well-characterized, classical species of which *Brucella melitensis* and *Brucella abortus* have the highest zoonotic potential. While other members of the genus are very similar on the genomic level, the zoonotic potential varies, and *Brucella ovis* is considered not zoonotic at all. Next to classical species, new emerging species are continuously being discovered and added to the genus, but the virulence and zoonotic potential of these identified species are largely unknown. Understanding these features is necessary to assess risk to the public and for the development of appropriate preventive and control measures.

To enable quick screening for the presence of virulence genes in *Brucella* species, we have developed a custom database to be used in an *in silico* pipeline based on the ABRicate tool (1). We used a two-step approach to identify virulence genes in *Brucella* species. The first step was to run ABRicate on the conservative set of well-defined virulence genes in the VFDB virulence database (2). This resulted in a maximum of 51 virulence genes that can be found in the classical *Brucella* species. Besides, we have created an additional database for ABRicate with 208 supplementary virulence genes, of which five genes have two distinct copies. This database comprising these supplementary virulence genes was named Bat-VFDB (Brucella addition to VFDB) and was based on *Brucella* virulence genes from literature and the Victors database (3–7). After gathering all gene sequences, the set was deduplicated based on identity, and for genes of other *Brucella* species that remained (11 *B. suis* and 15 *B. abortus*), homologous sequences from *B. melitensis* biovar 1 strain 16M were found using blastn to replace them within the final set. This *B. melitensis* strain was used, as this was the reference in most other papers concerning *Brucella* virulence genes, and homologs of multiple species present within the database were unwanted. Finally, this resulted in the full list of the 208 included genes in Bat-VFDB from the *B. melitensis* biovar 1 strain 16M (Table 1) (8).

**TABLE 1:** Genes included in the Bat-VFDB database^*a*^

B. mel. ORF	Locus tag in db	Gene	Putative or assessed function	Reference
BMEI0040	identical	xerD	Site-specific recombinase	Delrue et al. (4)
BMEI0082	identical	hpt	Purines synthesis	Delrue et al. (4)
BMEI0084	identical	lysA	Lysine synthesis	Delrue et al. (4)
BMEI0085	identical	Hypothetical protein	Brucella orphan gene	Delrue et al. (4)
BMEI0135	identical	omp19	Lipoprotein	Delrue et al. (4)
BMEI0157	identical	leuC	Valine and leucine synthesis	Delrue et al. (4)
BMEI0190	identical	pstP	Phosphoenolpyruvate phosphotransferase	Delrue et al. (4)
BMEI0258	identical	livH	Branched AA transport system	Delrue et al. (4)
BMEI0266	identical	pyc	Pyruvate carboxylase	Delrue et al. (4)
BMEI0267	identical	mosC	Rhizopine transport	Delrue et al. (4)
BMEI0271	identical	mgtA	Peptidoglycan	Delrue et al. (4)
BMEI0275	identical	mgpS	RNA helicase family	Delrue et al. (4)
BMEI0296	identical	purE	Purines synthesis	Delrue et al. (4)
BMEI0357	identical	asnC family	Transcriptional regulator	Delrue et al. (4)
BMEI0358	identical	dut	Pyrimidine synthesis	Delrue et al. (4)
BMEI0359	identical	macA	Macrolide efflux	Delrue et al. (4)
BMEI0382	identical	alkA	HhH-GPD superfamily base excision DNA repair	Delrue et al. (4)
BMEI0384	identical	dsbB domain	Disulfide bond formation protein	Delrue et al. (4)
BMEI0433	identical	dppA	Dipeptide uptake	Delrue et al. (4)
BMEI0451	identical	leuA	Leucine synthesis	Delrue et al. (4)
BMEI0455	identical	Hypothetical protein	Glutathione S-transferase, C-terminal domain	Delrue et al. (4)
BMEI0480	identical	pth	Peptidyl-t-RNA hydrolase	Delrue et al. (4)
BMEI0512	identical	trkH	Thioredoxin reductase	Delrue et al. (4)
BMEI0513	identical	lysR family	Transcriptional regulator	Delrue et al. (4)
BMEI0516	identical	aspC	Aminotransferase	Delrue et al. (4)
BMEI0526	identical	carAB	Glut. and pyr. synthesis	Delrue et al. (4)
BMEI0545	identical	pncA	Nicotinamide metabolism	Delrue et al. (4)
BMEI0605	identical	bicA	Macrolide efflux	Delrue et al. (4)
BMEI0615	identical	serB	Ser. synthesis	Delrue et al. (4)
BMEI0616	identical	miaA	IRNA synthesis	Delrue et al. (4)
BMEI0617	identical	ilvI	Val, Leu., Isoleu. synthesis	Delrue et al. (4)
BMEI0624	identical	ilvC	Val, Leu., Isoleu. synthesis	Delrue et al. (4)
BMEI0626	identical	aspB	Aminotransferase	Delrue et al. (4)
BMEI0671	identical	Hypothetical protein	TerC domain (efflux)	Delrue et al. (4)
BMEI0705	identical	cobB	Cobalamin synthesis	Delrue et al. (4)
BMEI0725	identical	thrA	Lys. synthesis	Delrue et al. (4)
BMEI0730	identical	gloA	Lactoylglutathione lyase (pyruvate metabolism)	Delrue et al. (4)
BMEI0781	identical	rpoA	RNA polymerase a- subunit	Delrue et al. (4)
BMEI0787	identical	recA	Recombinase A	Delrue et al. (4)
BMEI0827	identical	uppS	transglycosylase UPP synthetase	Delrue et al. (4)
BMEI0845	identical	ppiD	Rotamase D	Delrue et al. (4)
BMEI0867	identical	ntrY	Histidine kinase	Delrue et al. (4)
BMEI0872	identical	hfq	RNA-binding protein	Delrue et al. (4)
BMEI0876	identical	lon	Protease	Delrue et al. (4)
BMEI0898	identical	caiB domain	CAIB/BAIF family	Delrue et al. (4)
BMEI0933	identical	cysK	Cys. synthesis	Delrue et al. (4)
BMEI0979	identical	glnA	Glutamine synthase	Delrue et al. (4)
BMEI1040	identical	dsbA domain	Disulfide bond formation protein	Delrue et al. (4)
BMEI1043	identical	nifS	Nitrogenase cofactor synthesis protein nifS	Delrue et al. (4)
BMEI1056	identical	amiC	Cell-wall hydrolysis	Delrue et al. (4)
BMEI1060	identical	dsbA domain	Disulfide bond formation protein	Delrue et al. (4)
BMEI1069	identical	tig	Trigger factor	Delrue et al. (4)
BMEI1104	identical	arti	Arginine transport system	Delrue et al. (4)
BMEI1116	identical	cbbE	Ribulose-phosphate 3-epimerase	Delrue et al. (4)
BMEI1127	identical	purT	Purine synthesis	Delrue et al. (4)
BMEI1192	identical	glyA	Lys. degradation Ser. synthesis	Delrue et al. (4)
BMEI1229	identical	Hypothetical protein	Exonuclease X-T domain	Delrue et al. (4)
BMEI1237	identical	galE	UDP-glucose 4-epimerase	Delrue et al. (4)
BMEI1240	identical	purM	Purines synthesis	Delrue et al. (4)
BMEI1241	identical	purN	Purines synthesis	Delrue et al. (4)
BMEI1249	identical	omp25	Major OMP, omp3a	Delrue et al. (4)
BMEI1258	identical	BMEI1258	ABC transporter	Delrue et al. (4)
BMEI1281	identical	pyrC	Dihydroorotase	Delrue et al. (4)
BMEI1296	identical	spoT	ppGpp synthetase	Delrue et al. (4)
BMEI1301	identical	mosA	Inosamine methylase	Delrue et al. (4)
BMEI1330	identical	htrA	Protease	Delrue et al. (4)
BMEI1336	identical	feuQ	Histidine kinase	Delrue et al. (4)
BMEI1337	identical	feuP	Response regulator	Delrue et al. (4)
BMEI1339	identical	Hypothetical protein	Brucella/Agrobacterium orphan gene	Delrue et al. (4)
BMEI1361	identical	Hypothetical protein	NA	Delrue et al. (4)
BMEI1440	identical	dsbA domain	Disulfide bond formation protein	Delrue et al. (4)
BMEI1443	identical	Hypothetical protein	haloacid dehalogenase- like hydrolase domain	Delrue et al. (4)
BMEI1448	identical	Unknown function	EAL domain	Delrue et al. (4)
BMEI1450	identical	thrC	Thre. synthesis	Delrue et al. (4)
BMEI1468	identical	tldD	Putative modulator of DNA gyrase	Delrue et al. (4)
BMEI1488	identical	purF	Purines synthesis	Delrue et al. (4)
BMEI1498	identical	dxps	Thiamine synthesis	Delrue et al. (4)
BMEI1506	identical	aroC	Try. and ubiquinone synth.	Delrue et al. (4)
BMEI1513	identical	dnaJ	Chaperone	Delrue et al. (4)
BMEI1519	identical	purD	Purines synthesis	Delrue et al. (4)
BMEI1531	identical	Tetratricopeptide repeat family protein	Protein-protein interaction	Delrue et al. (4)
BMEI1553	identical	bacA	Peptide transport ?	Delrue et al. (4)
BMEI1606	identical	vsrB	Histidine kinase	Delrue et al. (4)
BMEI1611	identical	pyrD	Pyrimidines synthesis	Delrue et al. (4)
BMEI1636	identical	pgi	Glucose-6-phosphate isomerase	Delrue et al. (4)
BMEI1658	identical	Hypothetical protein	Brucella orphan gene	Delrue et al. (4)
BMEI1668	identical	hisD	His. synthesis	Delrue et al. (4)
BMEI1713	identical	malK	Maltose transport	Delrue et al. (4)
BMEI1742	identical	exsA	ABC transporter	Delrue et al. (4)
BMEI1759	identical	metH	Met. synthesis	Delrue et al. (4)
BMEI1766	identical	cysI	Sulfite reductase	Delrue et al. (4)
BMEI1786	identical	glnL	Nitrogen regulatory IIA	Delrue et al. (4)
BMEI1804	identical	glnD	ginB regulatory protein	Delrue et al. (4)
BMEI1809	identical	Unknown function	ERFK/YBIS/YCFS/YNH G family	Delrue et al. (4)
BMEI1844	identical	Hypothetical protein	Domain of unknown function (DUF930)	Delrue et al. (4)
BMEI1848	identical	ilvD	Val., Leu., Isoleu. synthesis	Delrue et al. (4)
BMEI1849	identical	cycY	Disulfide oxidoreductase	Delrue et al. (4)
BMEI1859	identical	Hypothetical protein	Uncharacterized protein family 0005	Delrue et al. (4)
BMEI1879	identical	Hypothetical protein	Brucella/ Mesorhizobium orphan gene	Delrue et al. (4)
BMEI1902	identical	Hypothetical protein	Brucella/Mesorhizobium orphan gene	Delrue et al. (4)
BMEI1915	identical	rpsA	Ribosomal protein S1	Delrue et al. (4)
BMEI1946	identical	mutM	Recovery from mutagenesis by alkylating agents	Delrue et al. (4)
BMEI2002	identical	dnaK	Chaperone	Delrue et al. (4
BMEI2041	identical	hisF	His. synthesis	Delrue et al. (4)
BMEII0017	identical	omp10	Lipoprotein	Delrue et al. (4)
BMEII0018	identical	hemH	Heme synthesis	Delrue et al. (4)
BMEII0039	identical	glt1	Glut. synthesis	Delrue et al. (4)
BMEII0040	identical	gltD	Glut. synthesis	Delrue et al. (4)
BMEII0052	identical	nodV	Histidine Kinase	Delrue et al. (4)
BMEII0056	identical	mgtB	Mg²* uptake	Delrue et al. (4)
BMEII0077	identical	dhbC	Siderophore synthesis, Fe³* uptake	Delrue et al. (4)
BMEII0089	identical	rbsK	Ribokinase	Delrue et al. (4)
BMEII0128	identical	Hypothetical Protein	Uncharacterized protein family (UPF0261)	Delrue et al. (4)
BMEII0136	identical	pheB	Dioxygenase	Delrue et al. (4)
BMEII0150	identical	fliC	Flagellin	Delrue et al. (4)
BMEII0151	identical	fliF	M-S ring	Delrue et al. (4)
BMEII0154	identical	motB	Flagellar motor	Delrue et al. (4)
BMEII0159	identical	flgE	Hook	Delrue et al. (4)
BMEII0166	identical	flhA	Export apparatus	Delrue et al. (4)
BMEII0177	identical	znuC	Zn2" uptake	Delrue et al. (4)
BMEII0178	identical	znuA	Zn2* uptake	Delrue et al. (4)
BMEII0274	identical	Unknown function	GTPase of unknown function, FeoB domain	Delrue et al. (4)
BMEII0300	identical	dbsA	Ribose transport	Delrue et al. (4)
BMEII0308	identical	cobW	Cobalamin synthesis	Delrue et al. (4)
BMEII0318	identical	Hypothetical protein	NA	Delrue et al. (4)
BMEII0336	identical	BMEII0336	ABC transporter	Delrue et al. (4)
BMEII0350	identical	dacF	Peptidoglycan synthesis	Delrue et al. (4)
BMEII0361	identical	araG (gguA)	L-arabinose transport	Delrue et al. (4)
BMEII0378	identical	fdhA	Formate dehydrogenase	Delrue et al. (4)
BMEII0428	identical	eryC	Erythritol metabolism	Delrue et al. (4)
BMEII0429	identical	eryB	Erythritol metabolism	Delrue et al. (4)
BMEII0485	identical	galcD	D-galactarate dehydratase	Delrue et al. (4)
BMEII0487	identical	nikA	Ni2" uptake	Delrue et al. (4)
BMEII0513	identical	zwf	Glucose-6-P dehydrogenase	Delrue et al. (4)
BMEII0527	identical	xseA	Exodeoxyribonuclease	Delrue et al. (4)
BMEII0570	identical	mocC	Rhizopine/inositol catabolism	Delrue et al. (4)
BMEII0573	identical	RpiR family	Transcriptional regulator	Delrue et al. (4)
BMEII0581	identical	sodC	Cu/Zn superoxide dismutase	Delrue et al. (4)
BMEII0584	identical	fbpA	Fe3* binding	Delrue et al. (4)
BMEII0591	identical	ugpA	Sn-glycerol transport	Delrue et al. (4)
BMEII0624	identical	ugpA	Sn-glycerol transport	Delrue et al. (4)
BMEII0626	identical	Hypothetical protein	Dipeptidase domain	Delrue et al. (4)
BMEII0659	identical	divK	Response regulator	Delrue et al. (4)
BMEII0669	identical	pyrC	Dihydroorotase	Delrue et al. (4)
BMEII0670	identical	pyrB	Pyrimidines synthesis	Delrue et al. (4)
BMEII0671	identical	aidB	Protection against alkylation damage to DNA	Delrue et al. (4)
BMEII0701	identical	rbsC	ABC transporter	Delrue et al. (4)
BMEII0759	identical	cydB	Cytochrome oxidase subunit II	Delrue et al. (4)
BMEII0761	identical	cydC	Cytochrome oxidase	Delrue et al. (4)
BMEII0762	identical	cydD	Cytochrome transport	Delrue et al. (4)
BMEII0799	identical	ssuB	Permease	Delrue et al. (4)
BMEII0823	identical	glpK	Glycerol kinase	Delrue et al. (4)
BMEII0881	identical	xfp	Lys. degradation	Delrue et al. (4)
BMEII0923	identical	BMEII0923	ABC transporter	Delrue et al. (4)
BMEII0931	identical	ndrI	Ribonucleotide reductase	Delrue et al. (4)
BMEII0932	identical	nrdH	Ribonucleotide reductase	Delrue et al. (4)
BMEII0935	identical	Hypothetical protein	Bacterial protein of unknown function (DUF89)	Delrue et al. (4)
BMEII0950	identical	narG	Nitrate reductase	Delrue et al. (4)
BMEII1001	identical	norE	Nitric oxide reduction	Delrue et al. (4)
BMEII1019	identical	caiB domain	CAIB/BAIF family	Delrue et al. (4)
BMEII1037	identical	Zinc Protease	Zinc protease	Delrue et al. (4)
BMEII1045	identical	Hypothetical protein	Haloacid dehalogenase-like hydrolase domain	Delrue et al. (4)
BMEII1053	identical	gluP	Glucose/galactose transport	Delrue et al. (4)
BMEII1066	identical	gntR family	Transcriptional regulator	Delrue et al. (4)
BMEII1084	identical	flgI	P-ring	Delrue et al. (4)
BMEII1093	identical	deoR family	Transcriptional regulator	Delrue et al. (4)
BMEII1101	identical	gtrB	Glycosyl transferase	Delrue et al. (4)
BMEII1116	identical	vjbR	Transcriptional regulator	Delrue et al. (4)
BMEII1124	identical	gnd	6-Phosphogluconate dehydrogenase	Delrue et al. (4)
BMEI1366	NA	bvfA	NA	Laviggne et al. (3)
BMEI0643	NA	ureD	NA	Sangari et al. (6)
BMEI0644	NA	ureG	NA	Sangari et al. (6)
BMEI0645	NA	ureF	NA	Sangari et al. (6)
BMEI0646	NA	ureE	NA	Sangari et al. (6)
BMEI0647	NA	ureC	NA	Sangari et al. (6)
BMEI0648	NA	ureB	NA	Sangari et al. (6)
BMEI0649	NA	ureA	NA	Sangari et al. (6)
BMEI0536	NA	omp28	NA	Simborio et al. (7)
BMEII0844	NA	omp31	NA	Sabri et al. (9)
BMEI0066	identical	NA	Two-component response regulator	Sayers et al. (5) and refs within Victors db
BMEI0080	BAB1_1988, BR1987	hisC	Histidinol-phosphate aminotransferase	Sayers et al. (5) and refs within Victors db
BMEI0101	BR1967	cysK	Cysteine synthase	Sayers et al. (5) and refs within Victors db
BMEI0156	BAB1_1906	rplS	Ribosomal protein L19	Sayers et al. (5) and refs within Victors db
BMEI0169	identical	gntR4	Transcriptional regulator, gntr family/aminotransferase class-i	Sayers et al. (5) and refs within Victors db
BMEI0233	BR1816	purH	AICARFT/IMPCHase bienzyme:methylglyoxal synthase-like domain	Sayers et al. (5) and refs within Victors db
BMEI0305	identical	gntR2	Transcriptional regulator, deor family	Sayers et al. (5) and refs within Victors db
BMEI0309	BAB1_1742	pgk	Phosphoglycerate kinase:G-protein beta WD-40 repeat	Sayers et al. (5) and refs within Victors db
BMEI0320	identical	gntR17	Transcriptional regulator, gntr family	Sayers et al. (5) and refs within Victors db
BMEI0430	identical	nolR	Nodulation protein	Sayers et al. (5) and refs within Victors db
BMEI0866	BAB1_1140, BR1117	ntrC	Nitrogen assimilation regulatory protein	Sayers et al. (5) and refs within Victors db
BMEI0878	BAB1_1128	uvrA	Exc. nuclease ABC subunit A	Sayers et al. (5) and refs within Victors db
BMEI0881	identical	gntR5	Transcriptional regulator, gntR family	Sayers et al. (5) and refs within Victors db
BMEI1019	identical	caiB	Molybdenum cofactor biosynthesis protein a	Sayers et al. (5) and refs within Victors db
BMEI1324	BAB1_0641, BR0617	pepN	Aminopeptidase N	Sayers et al. (5) and refs within Victors db
BMEI1573	identical	lysR18	Transcriptional regulatory protein, lysr family	Sayers et al. (5) and refs within Victors db
BMEI1905	BAB1_0034, BR0037	pheA	Prephenate dehydratase	Sayers et al. (5) and refs within Victors db
BMEI1913	identical	lysR13	Transcriptional regulatory protein, lysr family	Sayers et al. (5) and refs within Victors db
BMEI2000	BR2127	pmtA	Phospholipid N-methyltransferase	Sayers et al. (5) and refs within Victors db
BMEII0116	identical	gntR10	Transcriptional regulator, gntr family	Sayers et al. (5) and refs within Victors db
BMEII0158	identical	ftcR	Two-component response regulator	Sayers et al. (5) and refs within Victors db
BMEII0390	identical	lysR12	Transcriptional regulatory protein, lysr family	Sayers et al. (5) and refs within Victors db
BMEII0475	identical	gntR1	Transcriptional regulator, gntr family	Sayers et al. (5) and refs within Victors db
BMEII0559	BAB2_0513, BRA0727	gcvT	Glycine cleavage system T protein	Sayers et al. (5) and refs within Victors db
BMEII0561	BAB2_0515, BRA0725	gcvP	Glycine cleavage system P protein	Sayers et al. (5) and refs within Victors db
BMEII0625	BAB2_0585, BRA0655	ugpB	Glycerol-3-phosphate ABC transporter	Sayers et al. (5) and refs within Victors db
BMEII0695	BRA0572	pcs	Phosphatidylcholine synthase	Sayers et al. (5) and refs within Victors db
BMEII0894	BRA0354	oxyR	Transcriptional regulator OxyR	Sayers et al. (5) and refs within Victors db
BMEII0996	BRA0251	norD	norD protein	Sayers et al. (5) and refs within Victors db

^*a*^ “NA” indicates not available.

Running ABRicate with this database is the second step, which will result in a comprehensive list of known virulence genes present in one or multiple *Brucella* genomes. However, do consider this analysis only provides information about the presence of the genes, but not on their functionality or activity. For both steps, the default cut-off of 80% identity was used for a gene to be considered present or absent. Based on the total number of genes found, a first screen for potential virulence can be made in support of an initial risk assessment.

## Data Availability

The database and instructions on running a search through ABRicate are available on https://git.wur.nl/wbvr-projects/Bat-VFDB. Gene sequences were derived from the genome assembly of *B. melitensis* biovar 1 strain 16M with accession number AE008917.1 from GenBank https://www.ncbi.nlm.nih.gov/nuccore/AE008917.1/.
